# Structural and chemical insights on the incorporation of americium into zircaloy-derived monoclinic zirconia

**DOI:** 10.1038/s42004-025-01857-9

**Published:** 2025-12-26

**Authors:** Gabriel L. Murphy, Sara Gilson, Karin Popa, Damien Prieur, Sven M. Schenk, Sorin-Octavian Valu, Harry Ramanantoanina, Tim Prüßmann, Tonya Vitova, Kathy Dardenne, Jörg Rothe, Jean-Yves Colle, Olaf Walter, Nina Huittinen

**Affiliations:** 1 Institute of Fusion Energy and Nuclear Waste Management (IFN-2), Forschungszentrum Jülich GmbH, Jülich, Germany; 2 Institute of Resource Ecology, Helmholtz-Zentrum Dresden-Rossendorf, Dresden, Germany; 3 European Commission, Joint Research Centre (JRC), Karlsruhe, Germany; 4 Institute for Nuclear Waste Disposal (INE), Karlsruhe Institute of Technology, Karlsruhe, Germany; 5 Institute of Chemistry and Biochemistry, Freie Universität Berlin, Berlin, Germany

**Keywords:** Materials chemistry, Corrosion, Nuclear waste

## Abstract

Monoclinic zirconia (*m-*ZrO_2_) forms on the internal surface of nuclear fuel Zircaloy cladding, acting as a critical barrier against radionuclide release at the fuel-cladding interface. However, the incorporation of minor actinide elements like americium in *m-*ZrO₂ and resultant structural chemistry remains poorly understood. Using a combination of diffraction and high-resolution X-ray spectroscopic techniques, we have examined *m-*ZrO_2_ with 5 mol% Am doping. We show Am enters *m-*ZrO_2_ tetravalently, where its solubility is approximately 1.0 mol%, *m*-(Am^4+^_0.011(7)_Zr^4+^_0.989(7)_)O_2_, attributed to the large Am^4+^ cation, where excess Am, that is predominantly trivalent, adopts a C-type (Am^4+/3+^_1-x_Zr^4+^_x_)_2_O_3+x_ phase in space group *Ia*-3. The known reversible high temperature phase transformation of *m-*ZrO_2_ to tetragonal is further shown to be reduced from 1150 ^o^C to 1050 ^o^C via Am^4+^ incorporation. The investigation provides critical insight into the chemical reactivity and speciation of minor actinide elements with nuclear fuel cladding related *m-*ZrO₂.

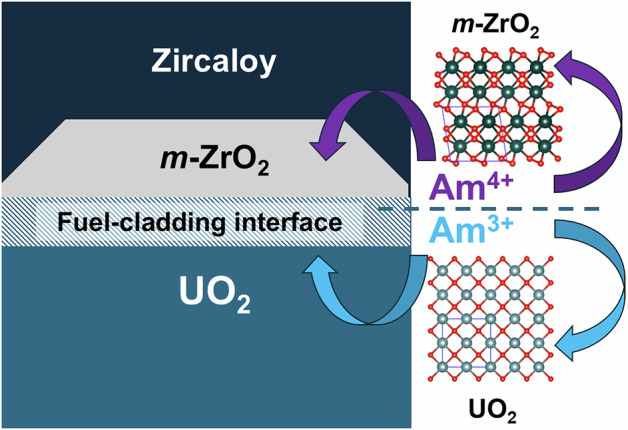

## Abstract (web summary)

Monoclinic zirconia (*m*-ZrO_2_) serves as a crucial barrier against radionuclide release in nuclear fuel Zircaloy cladding, yet the effects of minor actinide incorporation and chemical reactivity remain unclear. Here, the authors reveal that americium enters *m*-ZrO_2_ tetravalently with limited solubility and influences phase transformations, offering insights into behavior of minor actinides in spent nuclear fuel materials.

## Introduction

The chemistry of zirconia materials has been prolifically studied due to their superb properties, which have found application in a variety of topics ranging from advanced ceramics^1^, oxygen sensors^2^, optical fibres^3^ to fuel cells^4^, among others. Core to these applications is the well-defined phase relationships among the zirconia polymorphs, crystalline monoclinic, tetragonal and cubic, in addition to the amorphous phase^5^. The stabilities of these phases have been well established in literature to arise from factors including particle size, gas phase pressure, temperature, or doping level of specific elements^5,6^. Despite the exceptional literature available on *m-*ZrO_2_-based compounds and doping, a notable exception is the role minor actinides and 5 *f* element chemistry have in influencing the stability of zirconia materials. The relevance of this topic pertains to the formation of *m-*ZrO_2_ within the inner lining of spent nuclear fuel (SNF) Zircaloy cladding that forms during nuclear reactor operations and fuel burnup^7^. The interaction of the cladding and *m-*ZrO_2_ with the fuel is pertinent as it can affect the stability of the fuel elements during reactor operations and acts as a barrier to radionuclide release when stored as SNF^8^. The formation of ZrO_2_ within SNF is understood to occur from changes to the fuel’s oxygen potential, which is particularly influenced by the occurrence of Am^9^. This effect becomes more pronounced in higher burnup nuclear fuels and also mixed oxide (MOX) fuels that blend PuO_2_ and UO_2_^10,11^. Subsequently, Am and ZrO_2_ share an in-part symbiotic relationship within SNF, promoting the need for their joint chemical investigation, particularly regarding the incorporation and chemistry of the former in the latter.

Investigating the chemistry of minor actinide elements within *m*-ZrO_2_ has been chiefly performed via surrogate methods, often using lanthanide (*Ln*) elements. Relatively similar trends are observed between different trivalent *Ln*’s when doping *m-*ZrO_2_ due to their similar chemical properties^5^. In contrast, actinide elements exhibit a more diverse chemistry, which is influenced by the variability of 5 *f* electrons partaking in bonding; this, in turn, is then actinide element specific^12^. This is well exemplified by the chemistry of neighbouring Pu and Am, where the former can adopt oxidation states ranging from 3+ to 6+ in comparison to Am, which is typically confined to 3+ and 4 + ^13^. The difference is linked to the transition from itinerant to localised behaviour of the 5 *f* electrons in Pu and Am, respectively. Critically, this prevents direct extrapolation of chemical behaviour occurring for lighter actinide elements like Th, U and Pu to heavier and more difficult to handle minor actinide elements, including Am and Cm. Practically, Nd has been often used as a surrogate for Am in a variety of studies, due to similar chemical trends and ionic radii^14,15^. However, recent comparative studies have also highlighted associated shortcomings in inferring chemical behaviour through such surrogate studies of Am and Nd, especially as the lanthanide surrogate is not stable in the tetravalent oxidation state^16^. Consequently, in order to appropriately understand the chemistry of *m*-ZrO_2_ materials, particularly in the context of SNF stability, but also gauge the relevance of *Ln* surrogates like Nd, it demands direct studies utilising Am.

In considering the known Am doping chemistry of *m*-ZrO_2_, the understanding of the role of Am doping on the structural chemistry of *m*-ZrO_2_ is essentially not established. For instance, the level of solubility in the structure, the effect on stabilising the higher temperature tetragonal form, in addition to the overall redox chemistry, are not defined. Pertinently, there is no known Am-Zr-O phase diagram to help provide insight into these questions. If the chemistry of Nd is to be followed, previous work suggests the solubility is limited to 1 mol%, where after a defect fluorite structure is quickly precipitated, followed by eventual cubic pyrochlore structure in space group *Fd*-3*m* (No. 227)^17^. Similarly, if the chemistry of neighbouring Pu is to be followed, the solubility is found to be less than 1 mol% with a similar progression to an F-type cubic structure^18^. Critically, the ability of a specific element to be incorporated within *m*-ZrO_2_ has been shown to be highly dependent on its redox state, as has been shown previously for a variety of different elements^5^. At high temperatures, Am can readily disproportionate between its tri- and tetravalent states, depending on the oxygen potential present^19^. Simultaneously, *m*-ZrO_2_ is understood not to support oxygen defects that would occur through trivalent dopant incorporation, suggesting a preference for reaction with Am in its tetravalent state at high temperature. Subsequently, this suggests that during reactor operations, in pile partitioning and speciation may occur for different oxidation states of Am. However, this hypothesis has yet to be tested.

Considering what is described as a global nuclear renaissance in the use of nuclear energy, involving more advanced and higher burnup fuels^20–22^, the inevitable increase in pellet-cladding interactions will lead to enhanced fuel - *m-*ZrO_2_ interactions and subsequent solid-state chemical reactivity. Such phenomena will be considerably influenced by Am due to its contribution to changes in the fuel’s oxygen potential. However, the chemistry and ability of Am or other minor actinides to be incorporated within *m*-ZrO_2_ remain poorly established. Consequently, to develop critical and currently lacking fundamental insight, we have experimentally examined the incorporation of 5 mol% Am in *m-*ZrO_2_ to establish its chemistry and solubility. The synthesised material, produced via high temperature sintering under controlled oxygen potential conditions, was examined using X-ray diffraction (PXRD) with Rietveld analysis and Vegard’s law calculations to determine phase assemblage and assess Am incorporation within *m-*ZrO_2_, respectively. Am L_3_-edge and Zr K-edge X-ray absorption near edge structure (XANES) and extended X-ray absorption fine structure spectroscopy (EXAFS), as well as Am M_5_-edge high-energy resolution X-ray absorption near edge structure spectroscopic (HR-XANES) techniques, were used to examine both the redox states and local structural chemistry. The thermal dependence and stability of identified phases were further examined using high-temperature in situ powder X-ray diffraction. The results of this investigation are discussed in the context of existing *m-*ZrO_2_ literature, with comparison between Am and *Ln* incorporation, and with implications for fuel and SNF cladding chemical reactivity.

## Results and discussion

### Synthesis

The synthesis of 5 mol% Am-doped ZrO_2_ was conducted in a flowing Ar atmosphere at 1600 °C involving 1000 ppm H_2_O. At this sintering temperature and conditions, this resulted in an oxygen potential of −301 kJ/mol or 4 × 10^−9 ^atm based on readings from the input gas system used. When considering previous thermodynamic investigations of the Am-O system^19^, the conditions used suggest that Am is predominantly trivalent, existing as AmO_2-x_ prior to potential high temperature reactivity. Nevertheless, PXRD and spectroscopic analysis (discussed in subsequent sections) indicated that *m-*ZrO_2_ incorporates Am^+4^ at ~20% of the original 5 mol% Am used, i.e., ~1 mol%. According to the oxygen potential measurements of AmO_2_ by Otobe et al., this would still correspond to sub-stoichiometric AmO_2_ conditions, which would not typically favour such amounts of Am^+4^ when present solely as the binary Am oxide^19^. Notably, it has been shown that water vapour can interact strongly with oxide surfaces at high temperature, enhancing oxygen exchange or diffusivity among phases, particularly promoting the formation of thermodynamically stable structures^23,24^.

### Structural and spectroscopic characterisation

**Fig. 1:**
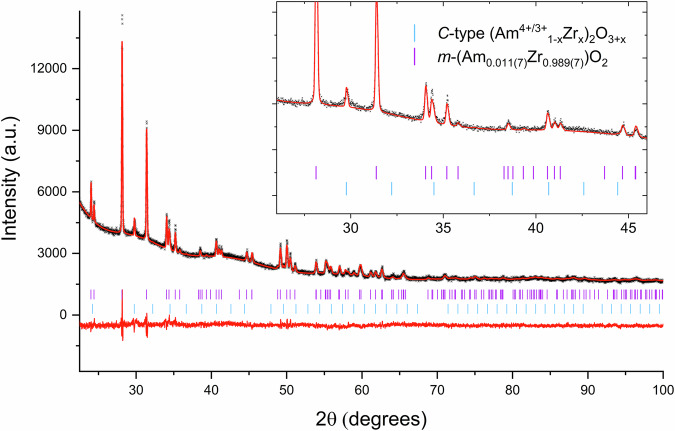
Rietveld profile made against PXRD data collected on synthesised 5 mol% Am-doped ZrO_2_. The black markers, upper and lower red lines respectively represent collected data, refined model and the difference curve. The vertical purple and blue markers respectively represent the (Am^4+^_0.011(7)_Zr^4+^_0.989(7)_)O_2_ monoclinic (SG *P*2_1_/*c*) and C-type (Am_1-x_Zr_x_)O_3+x_ (SG *Ia*-3) structures determined where their respective refined phase amounts are 98.15(6) and 1.85(6)%. Aberrations in the background are associated with the resin holder that the sample was mounted in. Rwp (%) = 1.95% and Rp (%) = 2.70%. Justification and detail for the final determined structures are provided in the section “*Phase Composition Determination via Vegard’s Law”*.

Collected PXRD data on the 5 mol% Am-doped ZrO_2_ sample was analysed using the Rietveld method, where a monoclinic structure in space group *P*2_1_/*c* consistent with *m*-ZrO_2_ could be well refined against the diffraction pattern. The Rietveld profile is provided in Fig. 1 with determined lattice parameters in Table 1. The lattice volume was determined to be 141.714(4) Å^3^, which is 1.063 Å^3^ larger than the expected value for pure *m*-ZrO_2_ of 140.651(3) Å^3^^25^. Such a lattice expansion is consistent with the inclusion of the Am cation within the crystal structure of *m*-ZrO_2_, due to its larger size both as a trivalent and a tetravalent cation (1.09 Å and 0.95 Å, respectively, for CN 8) in comparison to Zr (0.84 Å in CN 8)^26^.

**Table 1:** Refined lattice parameters for 5 mol% Am-doped ZrO_2_ for determined phases *m-*(Am_0.011(7)_Zr_0.989(7)_)O_2_ and C-type (Am_1-x_Zr_x_)_2_O_3+x_ in addition reference data for *m*-ZrO_2_^25^, C-type Am_2_O_3_^31^ and Am_2_Zr_2_O_7_^30^

Phase	*m-*(Am_0.011(7)_Zr_0.989(7)_)O_2_	*m*-ZrO_2_	C-type (Am_1-x_Zr_x_)_2_O_3+x_	C-type Am_2_O_3+x_	Am_2_Zr_2_O_7_
Reference	Present study	Gualtieri et al.^25^	Present study	Epifano et al.^31^	Belin et al.^30^
SG	*P*2_1_/*c*	*P*2_1_/*c*	*Ia*-3	*Ia*-3	*Fd*-3*m*
*a* (Å)	5.1626(4)	5.14604(6)	10.3914(18)	10.92(2)	10.66849(4)
*b* (Å)	5.2156 (2)	5.21162(7)	-	-	-
*c* (Å)	5.3314(4)	5.31308(7)	-	-	-
*β* (^o^)	99.1744(2)	99.222(1)	-	-	-
*V* (Å^3^)	141.714(4) Å^3^	140.651(3)	1122.1(6)	1302.171(5)	1214.252(4)

Justification and detail for the final determined structures is provided in the section “*Phase composition determination via vegard’s law”*.

In addition to the main phase *m*-ZrO_2_ structure, a minor secondary phase could be observed with reflections at ~30° and 34°. By considering the position of these reflections against possible phases that may occur in the Zr-Am-O system, it was determined that the best candidate matching phases would be a cubic structure in origin, particularly pyrochlore type in space group *Fd*-3*m* or C-type Am_2_O_3_ sesquioxide in space group *Ia*-3. Other potential phases, such as AmO_2_ or a secondary phase *m*-ZrO_2_ with depleted Am content, were ruled out based on the position of reflections. Subsequently, refinements were performed using pyrochlore and C-type Am_2_O_3_ sesquioxide type models to determine the origin of this secondary phase. To these phases, unit cell volumes of 1124.2(3) Å^3^ and 1122.1(6) Å^3^ were determined, respectively. Supplementary Information Note 1, provides details of the subsequent refinements performed. However, from the fitting values alone, it was not definitively clear which of these phases better describes the secondary phase occurring, although a more consistent fit could be observed with the C-type Am_2_O_3_ structure used.

### Am M_5_-edge high-energy resolution X-ray absorption near-edge structure spectroscopy

**Fig. 2:**
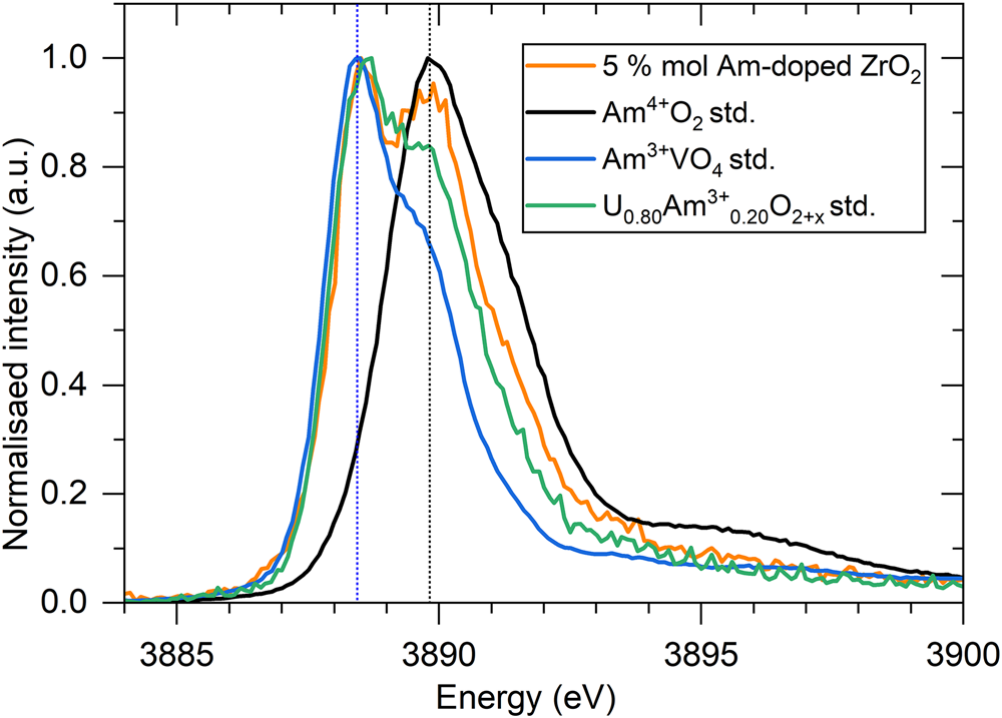
Am M_5_-edge HR-XANES spectra of the 5 mol% Am-doped ZrO_2_ sample. Am M_5_-edge HR-XANES spectra of the 5 mol% Am-doped ZrO_2_ sample performed on the Am M_5_-edge with the standards Am^4+^O_2_ and Am^3+^VO_4_, as well as U_0.80_Am^3+^_0.20_O_2−x_ (sintered)^28^. The vertical dashed blue and black lines correspond to the white line positions, representing the Am^3+^VO_4_ and Am^4+^O_2_ standards, respectively, which define the Am^3+^ and Am^4+^ oxidation state reference positions.

To understand the redox chemistry of Am within the 5 mol% Am-doped ZrO_2_ sample, and in particular the redox speciation between Am-rich and Am-poor phases in the sample, Am M_5_-edge HR-XANES experiments were performed. The normalised spectra are plotted in Fig. 2 in addition to the standards Am^4+^O_2_, Am^3+^VO_4_ and U_0.80_Am^3+^_0.20_O_2+x_^27^_._ The latter standard contains Am^3+^ incorporated into the UO_2_ lattice, with a slight trace Am^4+^ impurity on the surface, previously identified and discussed elsewhere^28^. This standard clearly shows that Am^3+^ in a cubic structure produces a double peak, with non-negligible peak intensity at the energy position of the Am^4+^ main absorption peak, i.e., at ~3890 eV. Comparing the 5 mol% Am-doped ZrO_2_ sample with the redox standards, clear contributions from both Am^4+^ and Am^3+^ can be seen (Fig. 2, orange traces). This is apparent from the peak intensity at 3890 eV, which is greater than in the U_0.80_Am^3+^_0.20_O_2+x_ standard, suggesting a predominance of Am^3+^ in the sample with a minor contribution of Am^4+^. The very similar shape of the standard and the Am-doped ZrO_2_ sample further suggests that the mixed Am^3+/4+^ is found within a cubic environment. Together with the clear predominance of Am^3+^, this supports the earlier PXRD results, suggesting that Am^3+^ resides in either a C-type Am_2_O_3_ or pyrochlore-type Am_2_Zr_2_O_7_ phase.

### Zr K-edge X-ray absorption near-edge structure and extended fine-structure spectroscopy

**Fig. 3:**
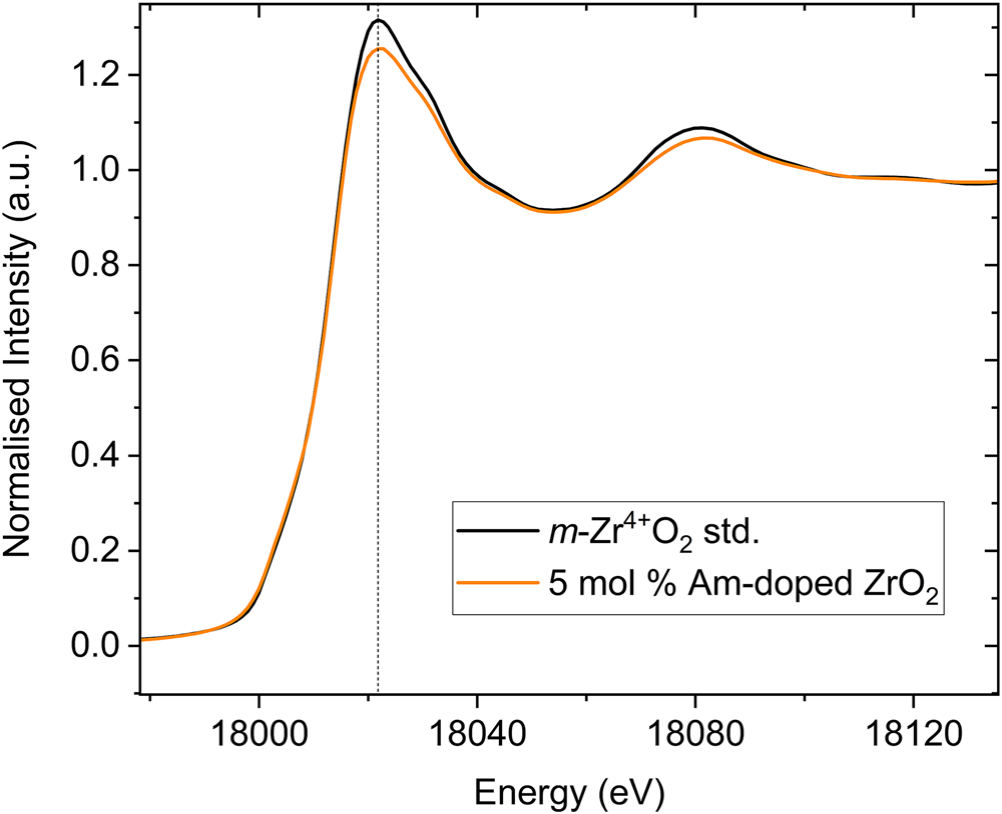
Normalised Zr K-edge XANES spectra of the 5 mol% Am-doped ZrO_2_ sample. Normalised Zr K-edge XANES spectra of the 5 mol% Am-doped ZrO_2_ sample with a *m-*Zr^+4^O_2_ standard. The vertical dashed black line corresponds to the white line position of the *m*-Zr^4+^O_2_ standard, representing the reference Zr^4+^ oxidation state.

Both Zr K-edge XANES and EXAFS analyses indicate that the local environment of the Zr atoms corresponds to a monoclinic structure. As shown in Fig. 3, the normalised Zr K-edge XANES spectrum of the 5 mol% Am-doped ZrO_2_ sample closely resembles that of a *m-*Zr^4+^O₂ reference. Furthermore, the Zr K-edge k^3^.χ(k) EXAFS spectrum was successfully fitted using a structural model based on the monoclinic phase. The structural parameters obtained from the fit (Table 2) are consistent with the monoclinic space group and suggest the incorporation of americium into the structure, as evidenced by slightly increased interatomic distances compared to pure *m-*ZrO₂^29^. Considering both the quality of the EXAFS fitting and the detection limits of the technique, the data support the conclusion that zirconium is present predominantly in the monoclinic ZrO₂ phase, adopting a distorted monocapped octahedral coordination environment. Subsequently, the Zr XANES and EXAFS analyses further indicate that the secondary phase is mainly americium-based.

**Table 2:** Structural parameters derived from the analysis of the EXAFS signal of the Zr K-edge for 5 mol% Am-doped ZrO_2_

Sample	Shell	R(A˚)	CN	σ^2^ (A˚^2^)
ZrK Rf = 1.5%	Zr–O	2.083 (5)	2	0.012 (1)
	Zr–O	2.176 (5)	3	0.011 (2)
	Zr–O	2.285 (5)	2	0.011 (2)
	Zr–M	3.52 (1)	7	0.007 (1)

### Am L_3_-edge X-ray absorption near-edge structure spectroscopy

**Fig. 4:**
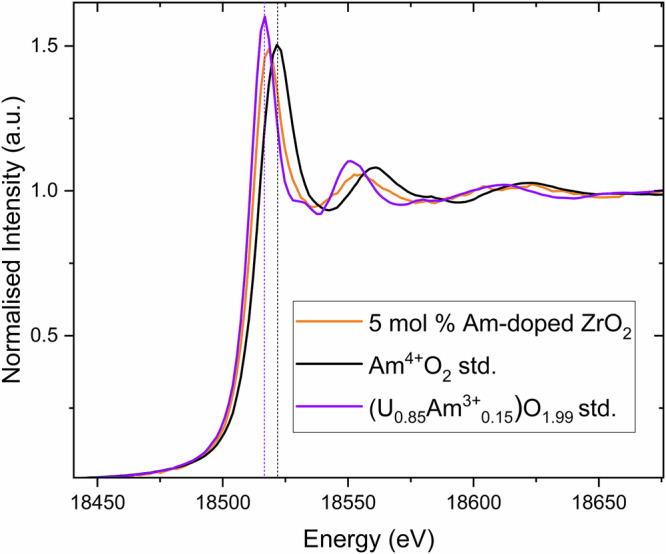
Normalised Am L_3_-edge spectra of the 5 mol% Am-doped ZrO_2_ sample. Normalised Am L_3_-edge spectra of the 5 mol% Am-doped ZrO_2_ sample measured with the standards Am^4+^O_2_ and (U_0.85_Am^3+^_0.15_)O_1.99._ The vertical dashed black and purple lines correspond to the white line position of the Am^4+^O_2_ and (U_0.85_Am^3+^_0.15_)O_1.99_ standards for the Am^4+^ and Am^3+^ oxidation states, respectively.

The oxidation state of Am within the 5 mol% Am-doped ZrO_2_ sample was investigated using Am L_3_-edge XANES spectroscopy. The choice of appropriate standards was guided by the M_5_-edge measurements. As shown in Fig. 4, the XANES spectrum of the 5 mol% Am-doped ZrO_2_ sample lies between those of Am^3+^ and Am^4+^ reference compounds, indicating the presence of mixed-valence states. Linear combination fitting reveals that approximately 80 ± 1% of the americium is present in the trivalent state, with the remaining 20 ± 1% in the tetravalent state. This result is in accordance with the qualitative fingerprint analyses of the Am M_5_-edge HR-XANES spectra. These results are displayed in Table 3, together with corresponding Zr data from all standard XANES measurements and related phase assignments discussed in subsequent sections.

**Table 3:** Summary of Am M_5_-edge HR-XANES, Am L_3_-edge and Zr K-edge XANES and EXAFS analyses of the identified Am and Zr chemical species

Identified chemical species	Local environment	Relative amount occurring	Assigned phase
Am^3+^	Cubic	80 ± 1%	C-type (Am^4+/3+^_1-x_Zr_x_)_2_O_3+x_
Am^4+^	Distorted monocapped octahedron	20 ± 1%	*m*-(Am^4+^_0.011(7)_Zr^4+^_0.989(7)_)O_2_
Zr^4+^	Distorted monocapped octahedron	99 ± 1%	*m*-(Am^4+^_0.011(7)_Zr^4+^_0.989(7)_)O_2_

The relative amounts of Am oxidation states were determined from linear combination fitting of the XANES data.

### Phase composition determination via Vegard’s law

From the experiments and analysis thus far, it is shown that the synthesis of 5 mol% Am-doped ZrO_2_ results in the formation of *m*-ZrO_2_ with an additional secondary phase in low amounts, in which the Am oxidation state is mixed, occurring as trivalent and tetravalent. Am L_3_-edge XANES analysis shows that 80% of the Am occurs as trivalent, and the remaining 20% as tetravalent. This result agrees with the results of the Am M_5_-edge HR-XANES analysis, which is more sensitive to small variations of the actinide oxidation states, but quantitative analyses are generally more difficult. From the Am M_5_-edge HR-XANES analysis, the coordination environment of the Am in the *m*-ZrO_2_ sample is found to be cubic. When the phase diagram of ZrO_2_ is considered^5^, *m*-ZrO_2_ is not expected to host oxygen defects, which would occur when Am^3+^ is incorporated within it. Rather, it is more expected Am^4+^ would incorporate into *m*-ZrO_2_ and the identified secondary phase accordingly contains Am^3+^. Based on the XANES analysis, it implies that of the 5 mol% Am that was used in the synthesis, only 1 mol% enters *m*-ZrO_2_ and it follows this is tetravalent Am. The subsequent 80% Am (4 mol%) is subsequently attributed primarily to trivalent and some tetravalent Am that reports to the secondary phase, which Rietveld analysis suggests is likely C-type Am_2_O_3_ or pyrochlore type Am_2_Zr_2_O_7_, i.e., an Am rich phase consistent with the HR-XANES and EXAFS. Invariably, the observed phenomena of phase separation is likely associated with the limited solubility of Am^4+^ within *m*-ZrO_2_. Subsequently, in order to identify the origin of the secondary cubic phase occurring whilst simultaneously determining the solubility of Am with *m*-ZrO_2_ and its composition, calculations were performed using Vegard’s Law (Eq. 1.) with determined lattice parameters from Rietveld analysis employed. The observed lattice expansion of the *m*-ZrO_2_ phase with Am^4+^ doping compared to the non-doped state will depend on the specific amount of Am that enters the lattice. Whereby, the observation of the secondary phases implies that the solubility limit has been reached in the main phase. Since Am was used in its tetravalent state during synthesis, further supported by XANES and particularly EXAFS analysis, the expansion of the *m*-ZrO_2_ structure can be assumed to follow a linear expansion via substitution of Zr^4+^ for Am^4+^ as given in Eqs. 1 and 2.

1 $$V={V}_{o}+x\cdot\Delta V$$

2 $${{Zr}}^{4+}1-{x{Am}}^{4+}x{O}_{2}$$

Using the determined lattice volume from the Rietveld method in Table 1 and comparing it to reference data from Gualtieri et al.^25^ provided also in Table 1, by Eq. 1, the necessary amount of Am^4+^ required to induce the observed 0.75% lattice expansion would be *x* = 0.0105 when using ionic radii of 0.95 Å and 0.78 Å for Am^4+^ and Zr^4+^, respectively, in CN = 7^26^. Accordingly, for the *m*-ZrO_2_ structure identified, this will correspond to an *x* value of 0.011(7) when the uncertainties of the ionic radii of Am and Zr are both considered, which corresponds to a formula of (Am_0.011(7)_Zr_0.989(7)_)O_2_, i.e., only 1.1(7) mol% incorporation into *m*-ZrO_2_. This value is considerably lower than the 5 mol% addition used in the synthesis, which will subsequently result in precipitation of a secondary Am-rich phase, as is described and observed. Notably, this determined value from Vegard’s law is consistent with the Am L_3_-edge XANES analysis and appears to corroborate both the amount of phase separation and also the difference in Am valence between the phases, namely *m*-ZrO_2_ contains only Am^4+^ and the secondary Am-rich phase is predominantly Am^3+^.

When the Zr K-edge XANES results are considered, they show that the Zr appears to be predominantly associated with the *m*-ZrO_2_ structure, with slightly increased interatomic distances compared to pure *m-*ZrO_2_ (Table 2). This corroborates the low amount of Am found in the monoclinic ZrO_2_ matrix and further implies that the secondary phase is likely very poor in the amount of Zr contained and rather rich in Am. Accordingly, it is suspected that the secondary phase is more likely C-type Am_2_O_3+x_ compared to pyrochlore-type Am_2_Zr_2_O_7_. Nevertheless, this can be further confirmed and understood when examining determined lattice parameters from Rietveld refinements, as was previously performed. For the pyrochlore-type Am_2_Zr_2_O_7_ a unit cell volume of 1124.2(3) Å^3^ was determined, this can be compared against the determined lattice volume of Am_2_Zr_2_O_7_ synthesised by Belin et al.^30^ of 1214.252(4) Å^3^. Since the unit cell volume of 1124.2(3) Å^3^ is considerably smaller than that determined by Belin et al., this would imply that the amount of Am with the pyrochlore structure is significantly less than the Am:Zr 1:1 ratio. Such an argument is difficult to support considering the noted significance ejection of Am from *m*-ZrO_2_, which should coincide with only some minor Zr incorporation. In the case of C-type Am_2_O_3+x_ a lattice volume of 1122.1(6) Å^3^ was determined, which is smaller than the reference C-type Am_2_O_3+x_ value provided by Epifano et al.^31^ of 1302.171 Å. For a C-type Am_2_O_3_ structure occurring as a secondary phase, it is likely that some Zr will be incorporated in the structure when it is ejected from the *m*-ZrO_2_ phase. Since XANES measurements show Zr occurs as Zr^4+^ this will lead to a contraction of the C-type Am_2_O_3_ structure, which is consistent with the trend in lattice parameters when the end-member value of Epifano et al.^31^ is compared to the solid solution value determined here. Naturally, variations in oxygen stoichiometry, although difficult to quantify, will further influence the lattice parameters when comparing the investigations; nevertheless, the results are still very consistent. Subsequently, it is argued that the significant excess Am that does not enter *m*-ZrO_2,_ results in the formation of a C-type (Am^4+/3+^_1-x_Zr_x_)_2_O_3+x_ structure where x is small and the Am exists primarily trivalently, but also in part tetravently, as supported by PXRD, HR-XANES, and standard XANES analysis. Using the determined chemistries of the phases from the analyses and applying quantitative phase analysis via the Rietveld refinement method indicates the *m*-(Am^4+^_0.011(7)_Zr^4+^_0.989(7)_)O_2_ and C-type (Am^4+/3+^_1–x_Zr_x_)_2_O_3+x_ phases occur as 98.15(6)% and 1.85(6)% respectively from the original synthesis of 5 mol% Am-doped ZrO_2_, whereby the former phase consists of 1 mol% Am as opposed to the latter phase that has 4 mol% from the total 5 mol% that was used. Interestingly, the amounts of Am deposited between the phases observed are strikingly consistent with what is seen in the Nd_2_O_3_-ZrO_2_ phase diagram^17^, suggesting in this instance there is good congruency between the behaviour of Am and Nd within ZrO_2_.

### High-temperature X-ray diffraction

**Fig. 5:**
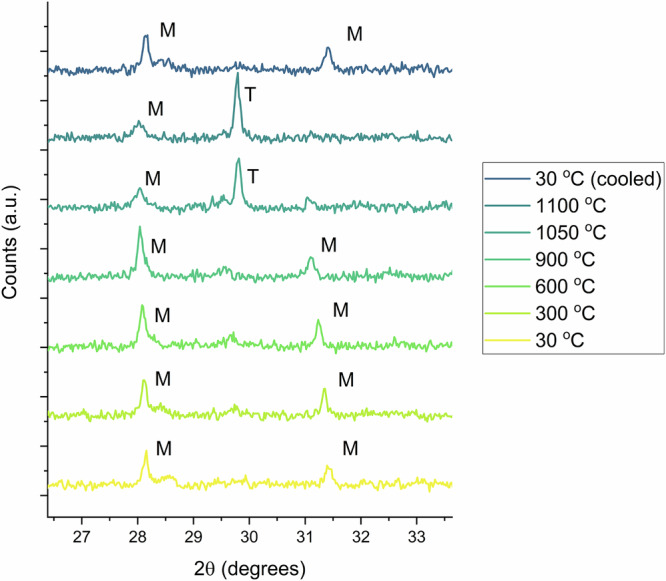
High temperature diffraction of 5 mol% Am-doped ZrO_2_. HT-PXRD of 5 mol% Am-doped ZrO_2_, where the transition from the *m*-(Am^4+^_0.011(7)_Zr^4+^_0.989(7)_)O_2_ phase (labelled M) can be observed to its tetragonal form (labelled T) reversibly between 900 and 1050 °C.

A notable known behaviour of *m*-ZrO_2_ is the ability to stabilise it in its tetragonal and cubic forms with increasing temperature, where the transition temperature can be reduced via doping. An appropriate test of confirming Am^4+^ incorporation within *m*-ZrO_2_ can be achieved subsequently through HT-PXRD measurements and showing a reduced phase transformation temperature with doping. Accordingly, HT-PXRD measurements were performed on a small amount of 5 mol% Am-doped ZrO_2_ material (15 mg) where the sample was heated sequentially to 1100 °C before cooling to RT. As shown in Fig. 5, which provides a portion of the collected data, the phase transformation of the tetragonal form from the monoclinic can be observed between 900 and 1050 °C. This is significantly lower than that of pure *m*-ZrO_2_, which transforms to the tetragonal polymorph at approximately 1150 °C^5^. This indicates that the inclusion of Am^4+^ within *m*-ZrO_2_ lowers the transition temperature as expected, while also corroborating its incorporation within the structure.

### Reactivity of Am and *m*-ZrO_2_ in Nuclear Fuel

**Fig. 6:**
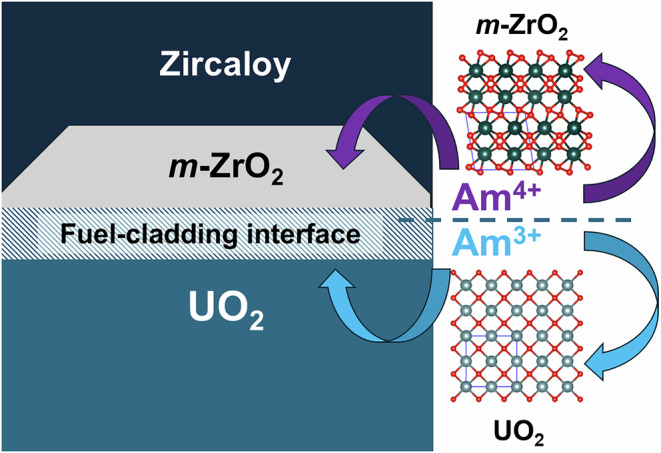
Schematic of reactions between Am redox states and *m*-ZrO_2_ and UO_2_ within Zircaloy cladded nuclear fuel at high burnup. Schematic of the inferred chemical interactions between *m*-ZrO_2_ and Am redox states at the UO_2_ fuel-Zircaloy cladding interface that can occur at higher fuel burnup, based on observations from this study.

From the results of the present investigation, it is pertinent to consider the reactivity and co-occurrence of *m*-ZrO_2_ and Am in UO_2_ based nuclear fuel. The *in-operando* formation of ZrO_2_ along the cladding inner surface of Zircaloy is attributed to the increase in the fuel’s oxygen potential that arises with increasing fuel burnup (fuel fission). Chemically, this effect originates from the accumulation of fission products, particularly those with lower valence than uranium (tetravalent). Lanthanides and minor actinides, which predominantly occur in the trivalent state, contribute strongly to this process. It is most pronounced at the rim of fuel pellets due to the known “rim effect”, arising from enhanced production and fission of Pu from ^238^U capture. As a result, oxidised phases can form in close proximity to the Zircaloy-based cladding, favouring the formation of oxidised ZrO_2_. Studies of SNF have identified that the ZrO_2_ polymorphs present are primarily *m*-ZrO_2_ and *t*-ZrO_2_^7,32^. The formation of *t*-ZrO_2_ is attributed to stabilisation by pressure, temperature, or significant incorporation of fission products^7,32^. In contrast, *m*-ZrO_2_ initially forms due to changes in the oxygen partial pressure and is therefore considered the first polymorph to form paragenetically within the fuel. Critically, *m*-ZrO_2_ tends to contact the UO_2_ fuel between the Zr cladding, which can promote solid-state reactions and chemical transport between the phases (Fig. 6)^32^. The present work has demonstrated that *m*-ZrO_2_ can retain Am^4+^ but rejects Am^3+^, which is soluble in UO_2_^28^. The redox state of Am at high temperature depends strongly on the oxygen partial pressure. In the present work, conditions of −301 kJ/mol or 4 × 10^−9 ^atm at 1600 °C were used. Previous studies indicate that under these conditions, Am^4+^ is not stable in binary oxides^19^. However, the presence of water vapour can enhance oxygen exchange or diffusivity of ions with other phases, provided it results in thermodynamic stabilisation^23,24^. In the present synthesis, 5 mol% Am was used, of which 20% reports to the *m*-ZrO_2_ phase, suggesting that tetravalent Am is stabilised within *m*-ZrO_2_. Such results on thermodynamic structure stabilisation have been made for other actinide oxide materals^33,34^. In relation to UO_2_-based nuclear fuel, comparable oxygen partial pressures used in the present study are generally only reached at higher burnup^35,36^. Furthermore, although high temperatures similar to those used in the present synthesis are generally not expected in nuclear fuels beyond accident scenarios, significantly lower temperature reactivity of *m*-ZrO_2_ can be expected^5^. Consequently, interactions between Am and *m*-ZrO_2_ can be expected at high burnup. The rejection of Am^3+^ from *m*-ZrO_2_ implies in pile chemical partitioning between the Am^4+^ and Am^3+^ redox states with the *m*-ZrO_2_ and UO_2_ phases, respectively. This process is graphically illustrated in Fig. 6. This chemical behaviour could consequently influence both the stability of the fuel cladding and the Am source term during SNF storage.

## Conclusions

The solubility and structural chemistry of 5 mol% Am doped *m*-ZrO_2_ has been determined through a combination of structure and spectroscopic techniques. Synthesis of a 5 mol% Am-doped ZrO_2_ is found to result in the formation of major phase *m-*(Am^4+^_0.011(7)_Zr^4+^_0.989(7)_)O_2_ and a minor phase that is attributed to a C-type (Am^4+/3+^_1-x_Zr^4+^_x_)_2_O_3+x_ structure, where x is small, as shown via PXRD, HR-XANES, XANES, EXAFS measurements and supported by Vegard’s law calculations. The known HT transformation from *m*-ZrO_2_ to *t*-ZrO_2_ occurring above 1150 °C is found to be reduced to below 1050 °C via HT-PXRD in situ measurements, due to the inclusion of the Am^4+^ cation in the lattice. The limited solubility of Am within *m*-ZrO_2_ is attributed to its size in its tetravalent form, the resistance of *m*-ZrO_2_ to incorporate lower valent Am, and the relative stability of trivalent Am at high temperature. Nevertheless, under the conditions used in this study, *m*-ZrO_2_ is able to successfully immobilise tetravalent Am, which would otherwise exist in its trivalent form. Considering the results of this investigation with the limited literature available on the Zr-Am-O system and the complete lack of a ZrO_2_-Am_2_O_3_ phase diagram, it appears that Am behaviour closely resembles that predicted by the ZrO_2_-Nd_2_O_3_ phase diagram. More broadly, given the symbiotic relationship between Am and ZrO_2_ within SNF, whereby the occurrence of Am during fuel burnup contributes to the formation of *m-*ZrO_2_ on the inner surface layer of Zircaloy cladding via elevation of the fuel’s oxygen potential^11^, the study provides key chemical insight into the interaction between these two species. Particularly, that *m*-ZrO_2_ appears to selectively incorporate Am^4+^ over Am^3+^. Conversely, this implies Am^3+^ would occur as binary oxide, soluble within the UO_2_ fuel matrix, which in turn creates an *in operando* partitioning effect between the redox states of Am with *m-*ZrO_2_ and UO_2_ in nuclear fuel during burnup. This consequently has repercussions for the stability of the Zircaloy cladding, whilst also for the distribution of minor actinides and Am within SNF assemblies.

## Experimental

### Synthesis

*m-*ZrO_2_ was synthesised following a high-temperature solid-state method. AmO_2_ was weighed out in a hot-cell glovebox and mixed with pre-acquired ZrO_2_ powder in stoichiometric quantities, targeting 5 mol% addition of Am. A dedicated and thoroughly cleaned working area within the hot-cell glovebox was used for the work, and all equipment required for the synthesis, including mortar with pestle, spatulas, crucibles and pellet dies, was either purchased new or cleaned with ethanol and water before synthesis. The AmO_2_ was used at the availability of the Joint Research Centre (JRC) Karlsruhe, Germany. The AmO_2_ was characterised prior to use regarding the presence of other actinides, of which maximum amounts of Pu and Np of 0.98(12)% and 3.47(4)% were determined from mass spectroscopy. The two reagents were homogenised using a mortar and pestle and ground into a fine powder. This powder was then compacted into a green pellet using a 5 mm diameter pellet die and a hydraulic press that applied 8 kN of force. Then, the pellet was transferred into a Mo crucible and placed in a furnace. The furnace was heated at a ramp rate of 200 °C/h to 1600 °C, held at 1600 °C for 48 hours, and then cooled at a rate of 200 °C/h. During sintering, a high-purity Ar atmosphere (Ar 98.998%, <4 ppb O_2_ and <3 ppb H_2_O) that contained 1000 ppm H_2_O was used. At sintering conditions, this provides a calculated potential of −301 kJ/mol or 4 × 10^−^^9 ^atm based on the gas flow mixture input into the furnace. The sintered ceramic pellet was then removed for subsequent analysis.

### Powder X-ray diffraction

To safely and routinely study the synthesised Am^4+^ incorporated *m-*ZrO_2,_ a piece of the sintered pellet was separated, ground to a fine powder and embedded in Loctite^®^ Double Bubble 2-part epoxy adhesive (5 minutes at 20 °C) resin matrix mounted to a standard powder X-ray diffraction holder. To minimise the effects of self-irradiation damage, particularly lattice swelling, the synthesised sample was measured within 1 day of synthesis. Measurements were performed using a Bruker D8 powder X-ray diffractometer using Cu Kα radiation (Kα_1_/Kα_2_–1.54056 Å/ 1.54439 Å, 40 kV, 40 mA, Ge(111) monochromator). Measurements were recorded from 15° to 100° 2θ in step sizes of 0.0017° with a counting time of 1 s using a LynxEye detector. Collected data was analysed using the Rietveld method as implemented in the programme GSAS-II^37^. The peak shapes were modelled using a pseudo-Voigt function, and the background was estimated using a 6–12 term shifted Chebyshev function. The atomic parameters of the phases examined were fixed based on determined chemistries. The scale factor, detector zero-point and lattice parameters were refined together with the peak profile parameters.

### High temperature X-ray powder diffraction

To study the thermal dependence of 5% mol Am-doped ZrO_2_, high temperature X-ray powder diffraction measurements (HT-PXRD) were performed on a Bruker D8 powder X-ray diffractometer using Cu Kα radiation (Kα_1_/Kα_2_–1.54056 Å/ 1.54439 Å, 40 kV, 40 mA, Ge(111) monochromator) and a LynxEye detector. Approximately 15 mg of sample was loaded on a Pt heating strip and placed in an Anton Paar HTK2000 heating chamber. A small amount of sample was loaded to minimise radiation exposure risks. The sample was heated from 30 to 1100 °C with the sample kept under vacuum conditions. The heating chamber was prior calibrated using a MgO standard over an extended temperature range, with a coefficient of thermal expansion consistent with previous works using the same diffractometer and heating chamber, determined and deemed acceptable^38^.

### Am M_5_-edge high-energy resolution X-ray absorption near-edge structure spectroscopy

For the determination of the oxidation states of Am present within the 5 mol% doped ZrO_2_ material, Am M_5_-edge HR-XANES experiments were conducted at the ACT station of the beamline for catalysis and actinide research (CAT-ACT beamline) of the KIT Light source at the Karlsruhe Research Accelerator (KARA)^39^. A Si(111) double-crystal monochromator (DCM) was used to monochromatize the incident beam. The beam was focused to 1000 × 1000 µm and further narrowed down by slits, leading to a sample spot size of ~500 × 200 µm (vertical × horizontal). HR-XANES spectra were acquired with a Johann-type X-ray emission spectrometer, using up to four Si(220) (Saint-Gobain, France) analyzer crystals with 1.0 m bending radius and an AXAS-M silicon drift detector (SDD, KETEK GmbH), which, together with the sample, were arranged in a Rowland circle geometry^39^. The spectrometer and sample were encased in a He glovebox to minimise the influence of atmospheric O_2_ and N_2_ during measurements. For the sample itself, a fragment of the sintered pellet was carefully mortared using ethanol. Approximately 1 mg of the finely ground powder was diluted in BN, pressed into a pellet, placed in a double containment and sealed twice with 13 µm Kapton foil. The HR-XANES spectra were measured with a step size of 0.1 eV from −5 to +20 eV from the white line (WL) of the respective edge and 0.5 eV in all other parts of the spectra. As no metallic reference foil with a suitable energy close to the Am M_5_-edge was available, energy calibration of collected spectra was done against an in-house Am^3+^-nPr-BTP reference sample. This was done by fixing the measured WL position of the reference sample to the known literature value for Am^3+^ (3888.5 eV)^27^. The spectra were thereafter normalised to have a WL intensity of close to one. Four spectra were averaged for 5 mol% Am-doped ZrO_2_. The results were compared against three standard samples, Am^4+^O_2_ as well as Am^3+^VO_4_ and U_0.80_Am^3+^_0.20_O_2+x_^27^ containing tetravalent and trivalent Am, respectively, for the determination of the Am redox state in *m*-ZrO_2_.

### Am L_3_- and Zr K-edge X-ray absorption near-edge structure and extended fine structure spectroscopy

Am L_3_- and Zr K-edge and XANES and EXAFS measurements of the 5 mol% Am-doped ZrO_2_ were conducted at the KIT Light Source at the INE-beamline^40^ using the KARA accelerator. Measurements were conducted at the Am L_3_- and Zr K-edges. The Larch software was used to extract EXAFS spectra from the raw absorption data^15^. Experimental Zr K-edge EXAFS spectra were Fourier-transformed using a Hanning window over the full k space range available 3.5–10.5 Å^−1^ Curve fitting was performed in k^3^ for *R* values in the range 1.2–4.3 Å. Phases and amplitudes for the interatomic scattering paths were calculated with the ab initio code FEFF8L^16^. Coordination numbers for cation-cation shells were fixed. Once satisfactory results were obtained, the constraints were removed with no significant variation. Linear combination fitting was used based on the WL absorption edge positions of the samples and the measured samples to quantitatively determine amounts and distributions of the investigated redox states present.

## Supplementary information

Supplementary Information PDF file

## Data Availability

The data that support the findings of this study are available from the corresponding author upon reasonable request.
