# Supplementary material for: Structural and chemical insights on the incorporation of americium into zircaloy-derived monoclinic zirconia
Source: Commun Chem. 2025 Dec 26;9:50. doi: 10.1038/s42004-025-01857-9 (PMC12847942; doi:10.1038/s42004-025-01857-9)
Supplement: Supplementary file 1 — Supplementary Information PDF file [file 42004_2025_1857_MOESM1_ESM.pdf]

Supporting Information

Communications Chemistry

Structural and Chemical Insights on the Incorporation of Americium into Zircaloy-Derived Monoclinic Zirconia

Gabriel L. Murphy[1],* Sara Gilson,[2] Karin Popa,[3] Damien Prieur,[2] Sven M. Schenk,[4] Sorin-Octavian Valu,[3] Harry Ramanantoanina,[4] Tim Prüßmann,[4] Tonya Vitova,[4] Kathy Dardenne,[4] Jörg Rothe,[4] Jean-Yves Colle,[3] Olaf Walter,[3] and Nina Huittinen[2,5]*

[1]*Institute of Fusion Energy and Nuclear Waste Management (IFN-2), Forschungszentrum Jülich GmbH, 52428 Jülich, Germany*

[2]*Institute of Resource Ecology, Helmholtz-Zentrum Dresden-Rossendorf, 01328 Dresden, Germany*

[3]*European Commission, Joint Research Centre (JRC), Karlsruhe, Germany*

[4]*Institute for Nuclear Waste Disposal (INE), Karlsruhe Institute of Technology 3640, D-76021 Karlsruhe, Germany*

[5]*Institute of Chemistry and Biochemistry, Freie Universität Berlin, 14195 Berlin, Germany*

*corresponding authors: Gabriel L. Murphy (g.murphy@fz-juelich.de), Nina Huittinen (n.huittinen@hzdr.de)

Supplementary Information Note 1. X-ray Powder Diffraction

Figure S1 displays Rietveld refinement profiles for 5 mol % Am-doped ZrO_2 where the secondary cubic phase has been analysed using (top) C-type cubic Am_2O_3 type and (bottom) pyrochlore cubic type $Am_2Zr_2O_7$ structures.

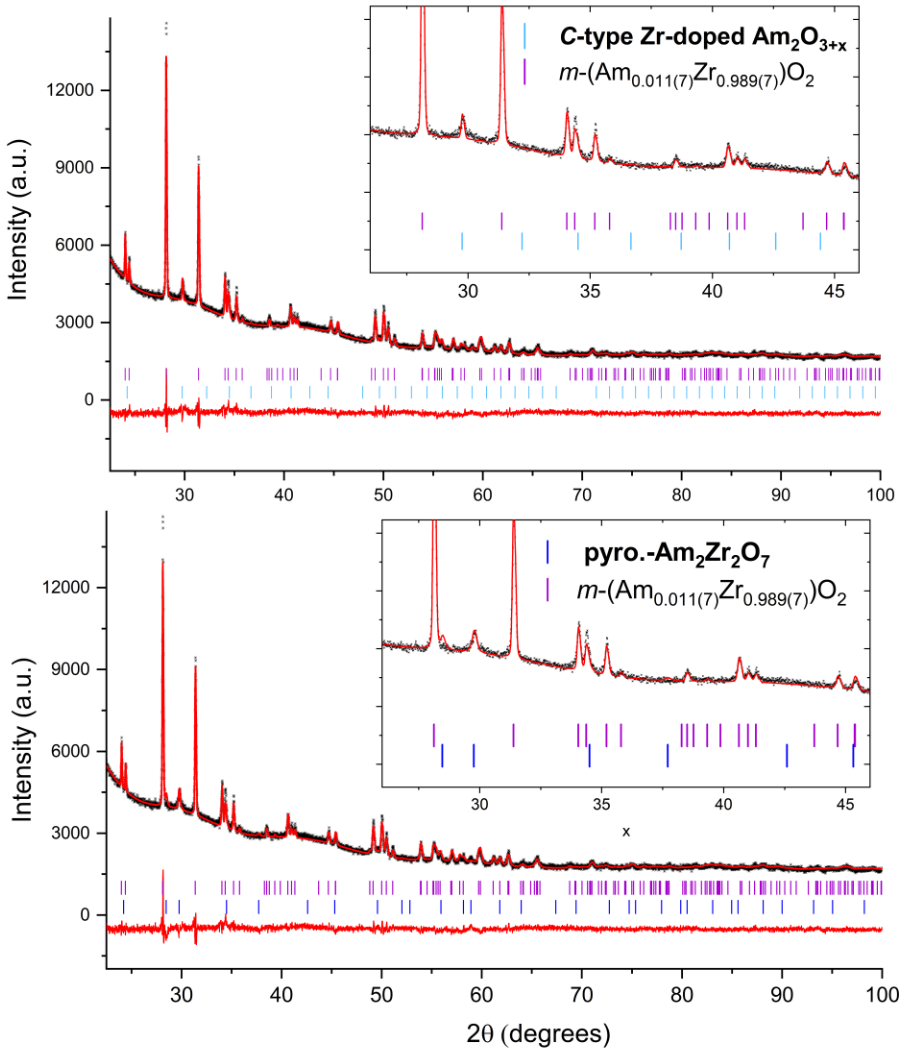

Figure S1. Rietveld fit of the PXRD data at ambient temperature for 5 mol % Am-doped ZrO_2 using consistent *m*-ZrO_2 monoclinic refinement models in addition to (top) C-type cubic Am_2O_3 type and (bottom) pyrochlore cubic type $Am_2Zr_2O_7$ structures. The black crosses, upper red line, lower red line and vertical purple/blue/cyan markers respectively represent observed data, calculated profile, difference profile and allowed reflections for the *m*-ZrO_2 (SG = $P2_1/c$), C-type Am_2O_3 (*Ia*-3) and pyrochlore-type $Am_2Zr_2O_7$ (SG = *Fd*-3*m*) respectively.
